# Switching between different cognitive strategies induces switch costs as evidenced by switches between manual and mental object rotation

**DOI:** 10.1038/s41598-024-56836-2

**Published:** 2024-03-14

**Authors:** Patrick P. Weis, Wilfried Kunde

**Affiliations:** Department of Psychology, Julius-Maximilians-Universität Würzburg, Lehrstuhl Fuer Psychologie III, Roentgenring 11, 97070 Wuerzburg, Germany

**Keywords:** Psychology, Human behaviour

## Abstract

Switching between tasks entails costs when compared to repeating the same task. It is unclear whether switch costs also occur when repeating the same task but switching the underlying cognitive strategy (CS). Here, we investigated whether CS switch costs exist despite overlap in mental processing between CSs and a lack of abstract goal (always “solve task X”) or answer key binding switches. Specifically, we asked participants to judge the identity of two misaligned objects by either mental or manual computer-mediated object rotation. In each trial of Block 1, to measure switch costs without choice-related cognitive processes, a cue indicated which CS (mental/manual) to use. In Block 2, the CS was freely chosen. Participants exhibited considerable CS switch costs for both cued and freely chosen switches. Moreover, Block 1 switch costs moderately predicted Block 2 switch frequency, while an overall tendency for CS repetition was observed. In sum, we found that switch costs are not confined to situations in which tasks are switched but generalize to situations in which the task stays identical and the CS is switched instead. The results have implications for modern computerized cognitive environments in which a multitude of cognitive strategies is available for the same task.

## Introduction

### Primer: cognitive strategies (CSs)

Typically, human agents can use various strategies to solve a problem. All different strategies include some cognitive processing and can thus be termed *cognitive strategies* (CSs). For example, preparing a meal can be based on accessing a memory-based recipe, a cookbook-based recipe, or continuously asking another person what to do next. Arithmetic can be done via mental arithmetic, memory recall, using a calculator, or asking another person ^e.g.,^^1–4^. Judging whether two misaligned objects are identical or not can be implemented by mental rotation^5^, bodily rotation by rotating the head^6^, or environment-based rotation by, e.g., turning a knob that rotates an object on a computer screen^7,8^, and so on. Thus, different CSs to achieve similar underlying goals exist and can be implemented by different means that include mental, body-related, or environment-related resources to varying degrees.

The existence of different CSs necessitates that the performer selects a specific CS for a given problem. Such CS selection processes have been at the core of many formalizations of the mind like rational or resource-rational analysis^9,10^ and been described as connecting Marr’s *computational level* with Marr’s *algorithmic level*^Figure 2 in^^10,11^. In other words, using different CSs for the same problem prominently changes the *algorithmic* and *implementational* levels^11^ whereas the abstract *computational* level^11^ stays identical.

Choosing between different CSs is related to but conceptually distinct from task selection. In task selection, such as when choosing between magnitude and parity judgments in voluntary task switching^12^, the problem itself changes, or in Marr’s^11^ terms, there is a change at the computational level. In CS selection however, problem and solution stay identical and only the *means* to obtain the solution differ. The present paper is focused on CS selection. Given the conceptual differences between task and CS selection, it is currently unclear whether what we know about task selection ^e.g.,^^13^ applies to CS selection as well. Yet, having more information about such transferability at hand would be desirable from an applied perspective: performers in a technologized world quite often need to select between an abundance of available CSs. In the present paper, we focus on whether the switch costs present for task switches transfer to CS switches.

### What are the costs of an abundance of CSs?

Conceivably, switching CSs is only feasible when multiple CSs are available. But it can be a struggle to maintain an overview of the available CSs. First, performances might differ between strategies. Most readers will be quicker solving “7 × 11 = ?” by relying on mental arithmetic or memory recall in comparison to consulting a calculator. Unfortunately, performance is not always that apparent. A conscientious problem solver would be required to monitor the performances of all relevant CSs for eventually being able to choose the best one. Although such monitoring can work well ^e.g.,^^6,14,15^ it certainly has its limits, especially if conflicting metacognitive information is available^16–18^ and is cognitively costly as indicated by the associated neural structures^19^ or the perceptual costs induced by error processing^20^. Second, keeping the relevant information for performing a certain strategy in the back of our heads—for example, where a calculator is located—can cause additional cognitive costs. Such costs multiply with more strategies at hand and are comparable to mixing costs in task switching^13^. Lastly, and most importantly for the present investigation, the mere switching forth and back between one and another strategy is likely to also cause costs when compared to sticking to one strategy, which is comparable to switch costs in task switching^13^.

In sum, having a multitude of CSs at hand for solving a cognitive problem could induce substantial costs. It is hard to imagine how a human performer can simultaneously monitor performances of several CSs, gauge the associated mixing and switch costs, flexibly switch forth and back between CSs, and still be able to focus on the task at the same time. This fact is recognized by literature that envisions the human problem solver as resource-limited rather than boundlessly optimal^10,21,22^. Thus, human performers might neither always strive nor be able to always make perfect choices. A good enough choice might sometimes be more practical than finding an optimal choice, which is known as *satisficing*^21,22^. Importantly, such good enough choices could significantly cut switch costs.

### Is self-chosen and/or cued preference for one CS beneficial?

One way to behave in a ‘good enough’ manner might be to choose one single CS and stick with it. Such behavior would be clearly beneficial if the chosen CS is performance-wise superior. But such behavior should also be beneficial if performances of different CSs are highly similar: The costs of performance monitoring, keeping the different CSs at the back of our heads, switching forth and back between them, and deciding which CS to use, could be offset by predominantly sticking to one strategy. In other words, if two CSs exhibit similar performances, it is likely beneficial to stick with one CS rather than to switch things up. Strong preference for one CS, sometimes with participants who exhibited no single CS switch—sometimes for more than 100 trials—was found with a variety of paradigms^15,23–27^^; also see^^28^.

That sticking to one CS is beneficial might be inferred from related research on voluntary task switching ^e.g.,^^12^. In voluntary task switching, participants are seeing one kind of stimulus but can choose whether to engage in task A (e.g., judging whether a number is smaller or bigger than five) or task B (e.g., judging whether a number is odd or even). In one study, an external cue indicated whether participants should repeat the same task with a new stimulus, or whether they should choose which task to perform^29^. Results indicated best performance for instructed (i.e., cue-based) repetition, medium performance for chosen repetition, and worst performance for chosen alternation. The performance drop from cued to freely chosen task repetitions suggests choice costs even if the task stayed identical between trials. The performance drop from chosen task repetition to chosen task switches also suggests switch costs, which seem to arise independently—unless participants omitted choice processing more frequently for repetition than for switch trials—of choice processes ^Experiment 6 in^^29^. In other words, in voluntary task switching, both freely chosen and cue-evoked preference for a specific task are beneficial. Furthermore, individual task switching costs seem to inform task preference, as indicated by across participants correlations between RT switch costs in a forced choice block and switch rate in a free choice block^30,31^.

### Differences between task switching and CS switching

**Figure 1:**
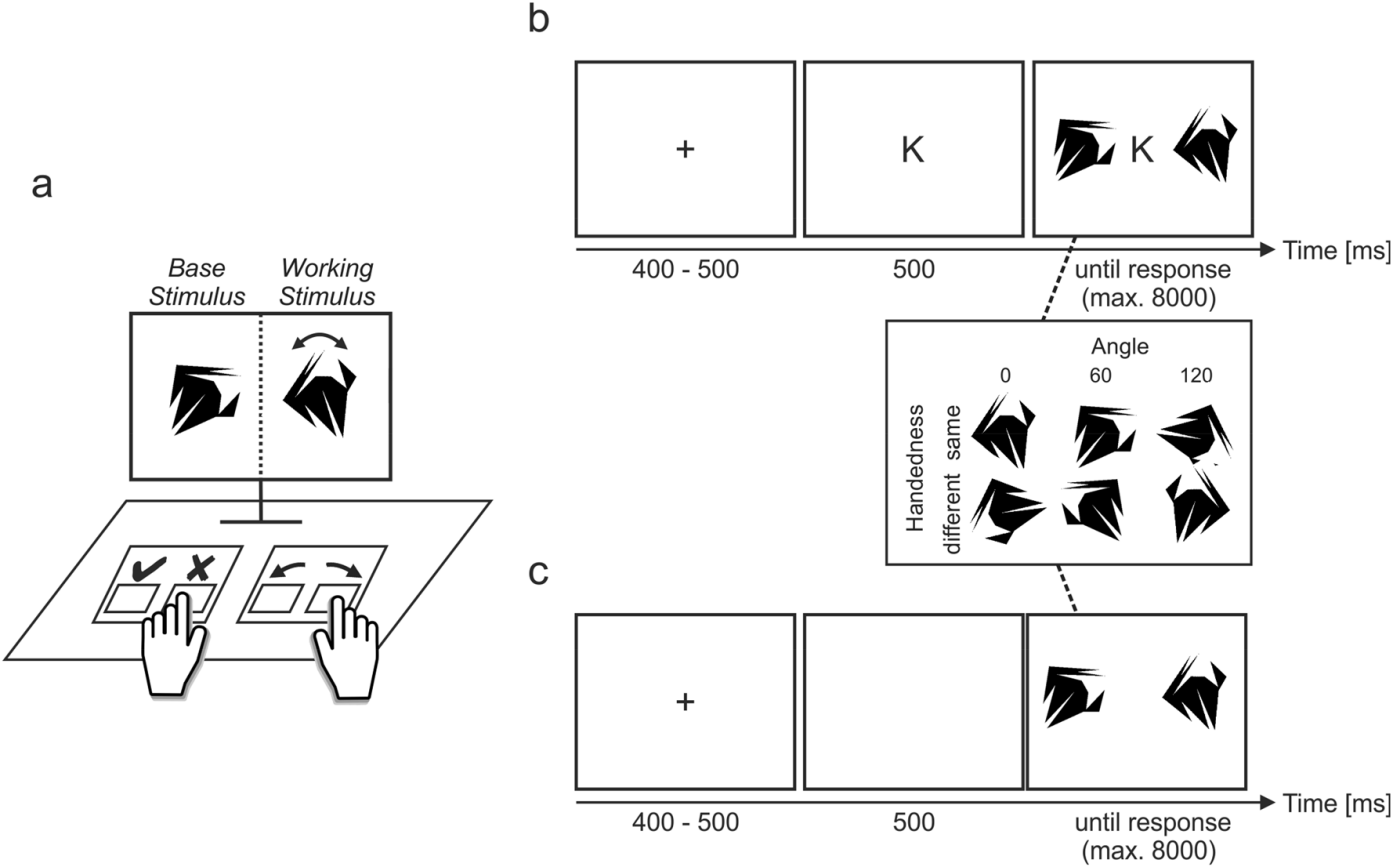
Extended rotation task. Note: in the extended rotation paradigm, participants compare the handedness of a base and a working stimulus. The base stimulus differs from the working stimulus in terms of angle and handedness. Participants need to indicate whether base and working stimulus are the “same” (only rotated in two-dimensional plane) or a “different” (first mirrored, then rotated in two-dimensional plane) stimulus by pressing the according key (same handedness is indicated by ✔ and was associated with the “a” key; different handedness is indicated by ✘ and was associated with the “s” key); (**a**) at the beginning of each trial with a specific shape, the working stimulus stays identical and the working stimulus is altered using the handedness and angle transformations. To help their decision, participants are able to offload their *mental rotation* process onto a physical interaction with the keyboard that affords rotating the working stimulus on screen (*manual rotation*).The coupling between rotation keys and working stimulus can replace a mental rotation process and thus constitutes an *extended cognitive strategy*, in contrast to the mental *internal cognitive strategy*. (**b**) A trial starts with a blank screen with a fixation cross for 400 to 500 ms, followed by a cue indicating which strategy to use (“M” for internal strategy, “K” for extended strategy) for 500 ms. Then, stimuli are presented for 8000 ms or until a response is given. If no response is given, the stimuli vanish and a text appears asking the participants to answer now—this text vanishes once a response is given. The paradigm was adjusted based on a similar paradigm used in previous research^8,15,43^. Block 2 trials have the same timing as shown in (**b**) but without any cues; (**c**). Note that practice trials additionally have feedback that is not shown in (**b**) or (**c**). Also note that during some trials, manual rotation would fail and an error is shown instead of the rotated stimulus (suppression; see main text for details). Here, the correct answer for the problem presented in (**a**) would be ✔ because rotating the working stimulus 60 degrees clockwise will result in the base stimulus.

The aim of the present manuscript is to investigate whether these benefits of strong preference for one task generalize to how we use cognitive strategies. That is, the target of the present manuscript is not the repetition of *tasks*, which implies the existence of different problems (e.g., either judging magnitude or parity). Instead, the target is the repetition of different CSs while keeping the problem the same (here, comparing the handedness of two stimuli via rotation; compare Fig. 1). Whether empirical differences between task switching and CS switching are predicted depends on how ‘task’ or ‘task set’ is defined, and there appears no clear consensus at present ^e.g.,^^32,33^. So we acknowledge that a “difference between task switching and CS switching” could equate to a “difference in task sets”, depending on conceptual preferences. In what follows, we more clearly delineate what we perceive as core differences between task switching and CS switching.

Despite similarities between task and CS switching, there are differences that render the study of CS switch costs worthwhile. First, for CS switching, costs related to switching the abstract goal of a task or digesting different types of stimuli fall away. Instead, abstract goal (here: rotating a stimulus and evaluating its handedness) and stimuli (here: 2D shapes) stay similar across trials; compare Fig. 1. In cued task switching, even if stimuli stay identical and only tasks are switched between trials (e.g., either judging a number’s magnitude or parity), switch costs of around 100 ms are common^34^. Thus, switch costs can be at least partially attributed to task/goal changes, which renders it questionable whether similar costs emerge for CS switches.

Second, due to the different cognitive resources used by different CSs, some CSs could even be viably used in parallel without inducing additional costs. In other words, strict use of one CS at a time might be hard to enforce and the resulting concurrent processing might reduce switch costs. That different CSs are indeed used in parallel has been suggested for two internal CSs^1,35^ as well as for internal and extended CSs^3,26^^; but also see^^28^. In contrast, for task switching, concurrent processing of two tasks (e.g., processing a number according to parity and size) appears both taxing, and in most cases also to be nonsensical because responses are needed for one task only, and responses in one task do not predict responses in the respectively other task. For the sake of completeness, we want to note that some concurrent dual-tasking paradigms do allow researching meaningful simultaneous processing of two tasks^36,37^. By contrast, the use of an internal CS (like mental object rotation) concurrently with an extended CS (like manual object rotation) appears much easier to do. Regarding object rotation, synergistic processing that is beneficial for different CSs could be due to rotation-specific priming of mental rotation by concurrent manual rotation^7,38^ as well as priming of manual rotation by concurrent mental rotation^39^.

Third, the correct response derived from one CS is identical to the response derived from the respectively other CS: both CSs are used to answer the question whether the two objects have the same handedness. In comparison to task switching, such response congruency might reduce interferences like response conflict. In sum, these considerations highlight differences between task and CS switching and suggest that alternating between different CSs could be easier than alternating between different tasks. Accordingly, here, we investigate whether findings from the task switching literature transfer to CS switching. In the absence of any contradictory evidence we assume this transfer to occur.

### Hypotheses

In the present study, we aim to validate the idea that choosing one CS and sticking with it—in comparison to switching forth and back between different CSs—does come with performance benefits. In particular, we hypothesize the following:

- **H1** There are CS switch costs. When a participant changes the CS used to solve a task from one trial to the next, performance drops as compared to CS repetition.
- **H2** The naturally occurring tendency to stick to one *task* ^‘repetition bias’ in^^12^ allows participants to avoid switch costs^31,40^. Analogously, sticking to one *CS* would allow participants to avoid switch costs if H1 can be confirmed. If participants are sensitive to switch costs, the size of individual switch costs should also drive a participant’s tendency to stick to one CS. The sensitivity would be indicated by a negative correlation of switch costs with switch frequency across subjects.

## Methods

### Participants

A total of 108 participants (mean age 37.5 years; age range 19–70 years; 2 diverse, 47 female, 58 male, 1 preferred not to answer) that fulfilled the inclusion criteria were measured in accordance with the preregistration protocol. The sample was recruited from several countries with English as main language, including the United States, Canada, and the United Kingdom, through the platform Prolific (www.prolific.co). The sample size is based on a power calculation for a *R*^2^ increase in linear multiple regression in G*Power ^version 3.1.9.2,^^41^; alpha = 0.05, 1—beta = 0.9, small to medium effect size *f*^2^ = 0.1. From the complete sample of 142 participants, 34 were excluded because they were (a) below 75% task accuracy across Blocks 1 and 2 (28 participants), (b) outside of 2.5 standard deviations around the sample’s RT mean (2 additional participants; only participants with at least 75% task accuracy were used for calculating RT outliers), (c) did not use the keyboard in at least 75% of “K”-cued Block 1 trials (0 additional participants), or (d) did use the keyboard in more than 25% of “M”-cued Block 1 trials (0 additional participants). Criterions (c) and (d) were necessary to ensure participants followed instructions and consequentially to ensure that the calculation of switch costs is meaningful. However, participants that fulfilled these exclusion criteria had already been excluded due to low accuracy. In addition to the preregistered protocol, we excluded 4 participants with a screen refresh rate below 15 Hz because the fidelity of keyboard-based rotation drastically drops below that rate.

### Apparatus

Participants completed the task on their personal computer running either Windows, Linux, or macOS. The task was programmed using PsychoPy, version v2021.1.4^42^, and presented online via the Pavlovia platform (www.pavlovia.org). Screen frame rates ranged between 18 and 165 Hz.

### Procedure and task

**Figure 2:**
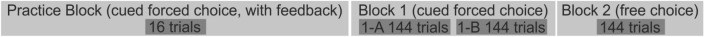
Procedure: rotation trials. Note: participants were able to redo the practice block if desired. For more information, please consult the main text.

After giving informed consent and answering basic demographic questions, participants read instructions and started working on trials of the Extended Rotation Task (Fig. 1). Specifically, participants engaged in 16 cued (forced) choice practice trials, 288 cued choice Block 1 trials (subdivided into Block 1-A and Block 1-B), and 144 free choice Block 2 trials (Fig. 2) without cues. Note that trial count was increased with respect to the preregistered procedure to avoid overly noisy estimates within participants. The 144 trials (36 stimuli × 2 angles × 2 handedness conditions) of Blocks 1-A, 1-B, and 2 were identical and presented in randomized order within each block. Note that the high number of Block 1 trials allowed us to rather precisely estimate cued switching costs, which we then used to predict Block 2 switches. We decided for a lower number of Block 2 than Block 1 trials because switches, in comparison to RT* switch costs, are less affected by outliers and thus easier to measure based on fewer trials. The difference of cued “choice” and non-cued free choice trials is illustrated in Fig. 1b, c, respectively, and further described in the corresponding figure caption. At the end of the study, participants were asked whether they experienced any technical problems, how frequently they thought about the decision between *mental* and *manual* rotation (for definition, consult caption of Fig. 1) in Block 2, how often they performed the mental and manual rotation in parallel in Block 1 and 2, respectively, whether they understood the instructions, and why they chose between mental and manual rotation in Block 2 the way they did.

During practice trials and during Block 1, trials were cued with the letters “M” or “K” (Fig. 1b). Participants were instructed to please only rotate mentally in front of the “mind’s eye” when seeing the letter “M” and only with the keyboard’s arrow keys when seeing the letter “K”. Whether an “M” or “K” cue was shown was determined right before each trial and independently of other trials. Both “M” and “K” had a probability of 0.5. This procedure prohibits savvy participants to extrapolate which cue might come up next. Even though we refer to both practice block and Block 1 as forced “cued choice”, participants could, in principle, choose to rotate without the keyboard even during trials with a “K” cue. However, whenever participants did commit such instruction violations, they received feedback that they did not obey instructions and were asked to please attend to cues further on. This type of feedback is given during practice trials only. Similarly, to help participants learn the task, accuracy feedback was given during practice trials only for 1000 ms following the response. Lastly, to help participants give fast answers, participants were asked to please speed up their responses whenever responses took longer than 4 s. This type of feedback was also given during practice trials only. After completing the practice block, participants were asked whether they understood the task and to repeat the practice bock if necessary: “If you understood all instructions, please [c]ontinue by pressing the "C" key. If you are unsure what to do and/or want to [r]edo the practice, please press the "R" key.”. After completing Block 1, i.e. at the beginning of Block 2, participants were instructed to now freely choose between manual and mental rotation. No cues were shown anymore (Fig. 1c).

One aspect is missing in Fig. 1’s trial depiction: for every trial, there was a 0.15 probability that keyboard-based rotation is suppressed. If an arrow key was pressed during these “suppressed” trials, the working stimulus vanished and the text “could not load the rotated image” appeared instead. This procedure was based on pilot data and necessary to adjust the performance of manual and mental rotation. Without the suppression procedure, pilot data showed that mental rotation accuracy would be substantially lower than manual rotation accuracy which would, in turn, substantially affect how participants choose between both rotation strategies in Block 2. Since we aimed to investigate the influence of switch costs on Block 2 strategy choice, such a strong contender for influencing choice would be undesirable.

### Stimuli

A total of 36 stimuli with sixteen edges was created based on a procedure described by Attneave and Arnoult^44^ and realized using a Matlab-based script provided by Collin and McMullen^45^. All stimuli were either presented as created by the script or rotated before presentation, depending on the condition (see Fig. 1b, c). All stimuli can be inspected at the linked online repository; an example stimulus can be inspected in Fig. 1.

### Analyses

#### Data cleaning

Practice trials were excluded from analysis. All trials with a reaction time (RT) of below 200 ms (0 trials) were excluded from analysis. All trials with RTs above 7900 ms (1.5% of all trials) were recoded to the value of 7900 ms. All suppression trials (see Procedure and Task section for definition) and the trials following suppression trials were excluded from analysis as well (27.2% of all trials). The very first trial after a break is also excluded (there is a break every 144 trials). Data cleaning followed the preregistered procedure.

#### Hypotheses-based analyses

The existence of switch costs (H1) was investigated using Block 1 trials and a 2 × 2 × 2 repeated measures ANOVA with the factors Angle (60°, 120°), Cue (“M”, “K”), and Switch (“yes”, “no”). RT* (RT of correctly answered trials divided by accuracy; also called Inverse Effiency^46^) was used as dependent variable (DV). RT* was used as main DV because switch costs can constitute both RT- and accuracy-related costs and RT* can capture both in a single value, which simplifies result presentation. Nevertheless, RT and accuracy were also analyzed separately for data transparency and exploratory purposes. A trial is considered a switch trial (Switch: “yes”) if the cue presented in the respective trial differed from the cue presented in the previous trial. A main effect of switch would confirm H1.

Whether the extent of individual switch costs drives an individual’s tendency to stick to one task (H2) was investigated with a hierarchical linear model using the percentage of switch trials during *Block 2* as DV (each participant has one value). All predictors were z-standardized for this analysis. As predictors, first, the absolute RT* difference between “K”-cued and “M”-cued Block 1 trials is entered to account for differential performance profiles of the internal and extended strategies (each participant has one value). Second, the switch costs were entered. Switch costs were calculated based on Block 1 trials (difference between RT* for switch trials and RT* for non-switch trials). If the incremental increase in explained variance from Step 1 to Step 2 is significant, H2 would be confirmed.

### Transparency and openness

We report how we calculated our target sample size, how we excluded data of entire participants but also partial data of participants, all manipulations, and all measures in the study. Data and Stimuli can be accessed in an online repository (see Data availability). Data were analyzed using R, version 4.1.1^47^ and the packages tidyverse (version 1.3.1), afex (version 1.0.1), and emmeans (version 1.6.3). Preregistration of the study’s design and hypotheses-driven analyses can be accessed in an online repository (see Data availability).

### Public significance statement

Humans can solve problems with different means: For example, objects can be rotated both in front of the mind’s eye and manually in the physical environment. Here, we show that switching between different means to rotate is associated with costs of around 100 to 200 ms. Considering such costs is important when solving problems with different means and when creating environments that are purposefully made to support problem solving.

### Ethics statement

Informed consent was obtained from all individual participants included in the study. This research complied with the tenets of the Declaration of Helsinki and was approved by the Ethics Committee at a European University.

## Results

### Sample descriptives

**Figure 3:**
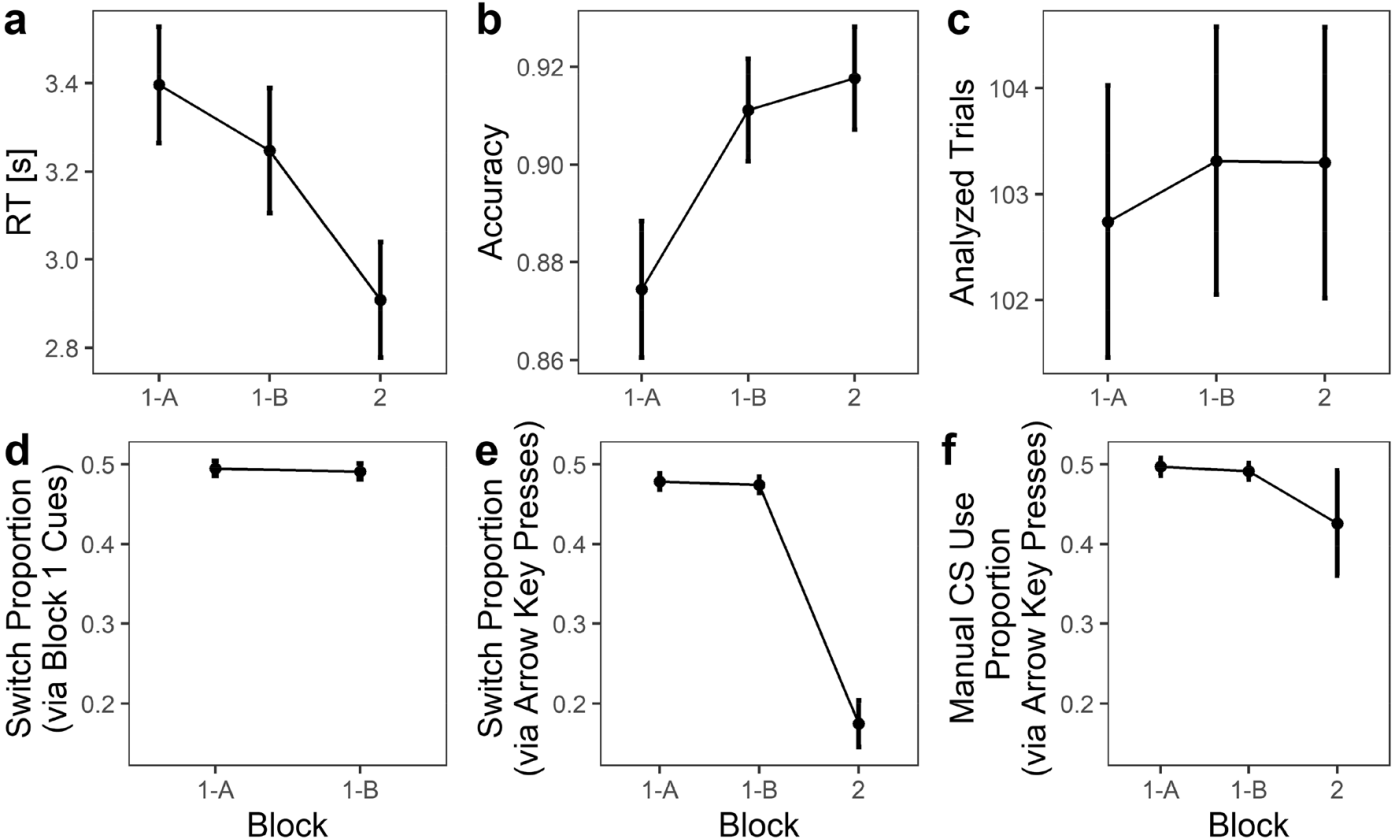
Sample descriptives. Note: error bars depict 95% CIs. *CS* cognitive strategy.

To get a crude overview of the present data, we briefly provide descriptives of some main DVs and how they develop over the course of Blocks 1-A, 1-B, and 2; Fig. 3. First, it should be kept in mind that reactions were rather slow in comparison to possibly easier tasks like magnitude estimation and without the availability of different CSs; Fig. 3a. For the present paradigm, RTs around 2.5 s have been reported for accuracy-oriented participants before^15^. The further increase in RT in the present study could be due to the higher age of the participants in comparison to earlier studies with the same paradigm. Second, accuracy was high; Fig. 3b. Third, out of the 144 trials in each block, on average 103 were analyzed in blocks 1-A, 1-B, and 2; see section Analysis: Data Cleaning for details on trial rejection. Fourth, switch proportion as indicated by differential cues (“M” or “K”) in Block 1 unsurprisingly mirrors the nature of cue probabilities (i.e., probability of 0.5 whether “M” or “K” was presented); Fig. 3d. To investigate CS switches in Block 2, we used an additional definition of switch trial: whenever trial n was solved *without* pressing an arrow key (which is used for manual rotation) and trial n + 1 was solved *with* pressing an arrow key (or vice versa), trial n + 1 was coded as a switch trial. In other words, this key-based approach defines a strategy switch based on actual behavior, namely a switch in whether arrow keys were used or not. Thus, a trial can count as a switch trial both if at least one arrow key was pressed in the switch trial (manual rotation) but no keys were pressed in the trial before (mental rotation) and if no keys were pressed in the switch trial (mental rotation) but at least one arrow key was pressed in the trial before (manual rotation). This key-based approach was necessary because no cues were given in Block 2. Fifth, as indicated by Fig. 3e, participants mostly followed cue-based CS instructions during Block 1 and switched less in Block 2. Interestingly, manual CS use proportion, on average, stayed nearly identical between Block 1 and 2, even though variance between participants substantially increased; Fig. 3f. Taken together, sample descriptives suggest high quality data that warrant a meaningful analysis of switch costs.

For full disclosure, we want to note that our calibration procedure with suppression trials was only able to partially adjust performances of manual and mental CSs. The manual CS was more accurate but slower while the mental CS was less accurate but faster. These differences might have decreased Block 2 switch rate due to a participant’s preferences regarding either speed or accuracy but should have otherwise not interfered with our hypotheses. Results are presented in more detail in the Supplemental Materials, Tables S1 and S2.

### Hypotheses testing: H1

**Figure 4:**
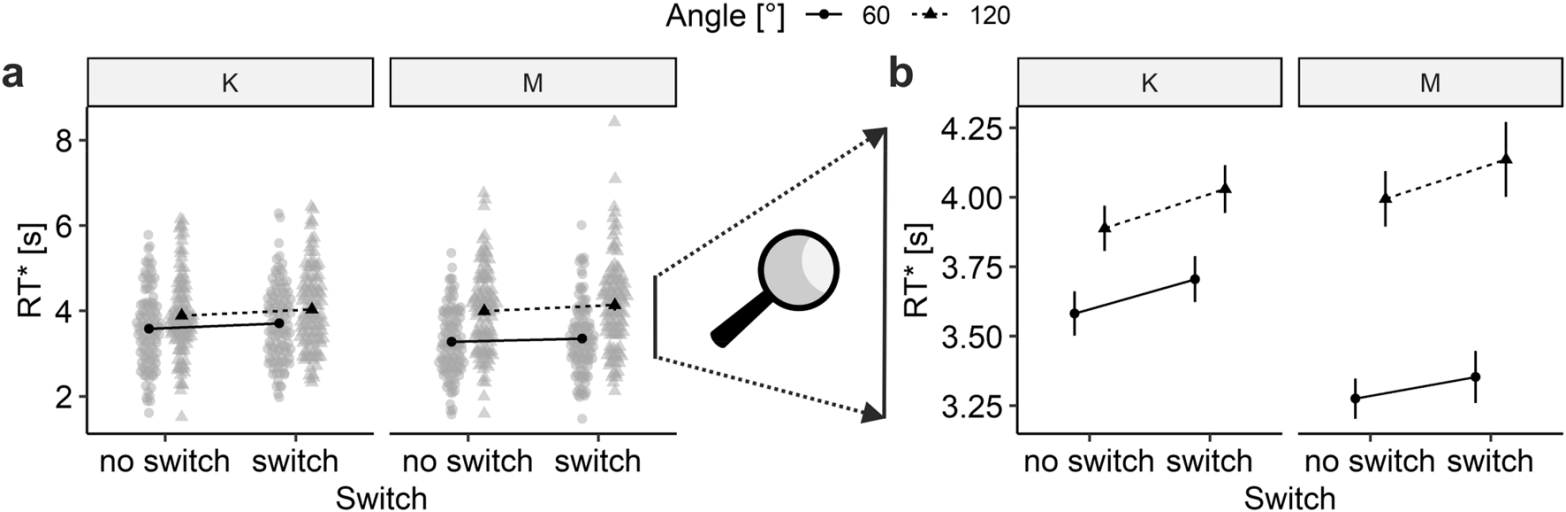
Switch costs in block 1 cued choice trials. Note: both plots depict the same grand averages either with (**a**) or without (**b**) single participant means for Block 1 trials split by cue (“K” or “M”). Error bars depict 95% within-participants error bars as implemented by R’s afex package (version 1.0-1). Please note that switch is defined by *cue* repetition or *cue* switch, not by actual participant behavior. However, participants with substantial incompliance to cues had been excluded prior to analysis (see section Participants) and both measures are highly similar (compare Fig. 3d, e).

RT* was affected by CS switches (*F*(1, 107) = 23.9, *p* < 0.0001, *η*_G_^2^ = 0.004). Switch trials were answered 121 ms (accuracy-corrected) slower than non-switch trials, which confirms H1. None of the interaction terms with *switch* were significant (all *p* > 0.4). RT* was also affected by angle (*F*(1, 107) = 271.1, *p* < 0.0001, *η*_G_^2^ = 0.077), cue (*F*(1, 107) = 4.4, *p* = 0.038, *η*_G_^2^ = 0.004), and the interaction between angle and cue (*F*(1, 107) = 56.7, *p* < 0.0001, *η*_G_^2^ = 0.014). The interaction indicates that rotation was quicker (i.e., the slope shallower) when using manual keyboard-based rotation (*M*_60°_ = 3643 ms, *M*_120°_ = 3959 ms) rather than mental rotation (*M*_60°_ = 3314 ms, *M*_120°_ = 4065 ms). Results are visualized in Fig. 4 and complete ANOVA results are presented in Table 1. Complementary analyses in which RT and accuracy were analyzed separately suggest that the RT* switch effect is driven by RT and not by accuracy (Tables S1 and S2, Supplemental Materials). The according RT effect is 103 ms.

**Table 1:** RT* ANOVA Results for testing H1 (block 1 trials only).

	DF1	DF2	*F*	*p*	η_G_^2^
Angle***	1	107	271.1	4.217 × 10^–31^	0.077
Cue*	1	107	4.4	0.038	0.004
Switch***	1	107	23.9	3.569 × 10^–6^	0.004
Angle × Cue***	1	107	56.7	1.717 × 10^–11^	0.014
Angle × Switch	1	107	0.5	0.479	< 0.001
Cue × Switch	1	107	0.2	0.678	< 0.001
Angle × Cue × Switch	1	107	0.2	0.692	< 0.001

***p < 0.0001; *p < 0.05.

### Hypotheses testing: H2

#### Dependent variable: RT*

Absolute RT* differences between K-cued and M-cued Block 1 trials did not predict Block 2 switch proportion, *R*^2^_adj_ = − 0.005, *F*(1, 106) = 0.47, *p* = 0.49. The situation did not change when adding the RT* switch costs between K-cued and M-cued Block 1 trials as a predictor, *R*^2^_adj_ = − 0.01, *F*(2, 105) = 0.30, *p* = 0.74; Table 2.

**Table 2:** Switch proportion regression results for testing H2: RT* (all participants).

Variable	Model 1				Model 2			
	Estimate	*SE*	*|t|*	*p*	Estimate	*SE*	*|t|*	*p*
Constant	0.175***	0.015	11.70	8.24 × 10^–21^	0.175***	0.015	11.66	1.17 × 10^–20^
**|**RT*_K-cued Trials_—RT*_M-cued Trials_|	− 0.010	0.015	0.69	0.49	− 0.011	0.015	0.70	0.49
RT*_Switch Trials_—RT*_Repetition Trials_					− 0.007	0.015	0.49	0.63
*R*^2^_Adj_	− 0.005				− 0.01			
Δ*R*^2^_Adj_					− 0.01			

Predictors are based on Block 1, DV on Block 2 trials, compare Fig. 2. The variance inflation factor for both Model 2 predictors is 1.001.

***p < 0.0001.

Given the hard boundaries of the DV at 0 and 1 and the substantial percentage of participants with 0 switches (23% of participants), we recognize that the present approach was not the optimal analytic approach and violates ordinary least squares (OLS) assumptions. However, we decided to report the present approach as it was preregistered and to additionally conduct complementary analyses using (1) an OLS analysis without participants that exhibited 0 switches to avoid the assumption violation that errors are not normally distributed and (2) a logistic regression to additionally account for boundary-related statistical issues.

In short, the full model with both predictors without participants that exhibited 0 switches (1) was still was not able explain more variance than the mean , *R*^2^_adj_ = − 0.01, *F*(2, 80) = 0.56, *p* = 0.57. The complementary logistic regression (2) indicated that switch costs predict switch proportion. However, due to overdispersion in the present sample (*Deviance/Degrees of Freedom* > *10* instead of *Deviance/Degrees of Freedom* ~ *1*), statistical significance was likely overestimated ^e.g.,^^48^. Hence, we conducted a quasi-binomial logistic regression which allows for modeling overdispersed data^49^. Similar to the previous analyses, deviance of the full model with two predictors was not significantly lower than the deviance of the null model without predictors; Step 1: df = 107, *χ*^2^ = 2178, Step 2: df = 105, *χ*^2^ = 2161, Δ*χ*^2^ = 17.2, *p* = 0.612.

#### Dependent variable: RT

Deviating from the main analysis plan for H2, but in accordance with reasonable data exploration that was also mentioned in the preregistration plan, we mirrored the previous analyses with RT rather than RT* as DV. We decided for this step because Block 1 switch trials were answered slower but not less accurate than non-switch trials (compare Tables S1 and S2). In other words, switches in the present study elicited RT costs but no accuracy costs. Focusing this analysis on RT could thus potentially eliminate accuracy-related noise while maintaining the main indicator of switch costs in the present study: RT.

That being said, absolute RT differences between K-cued and M-cued Block 1 trials did not predict Block 2 switch percentage, *R*^2^_adj_ = − 0.01, *F*(1, 106) = 0.31, *p* = 0.58. The situation did not change when adding the RT switch costs between K-cued and M-cued Block 1 trials as a predictor, *R*^2^_adj_ = 0.01, *F*(2, 105) = 1.61, *p* = 0.20; Table 3. However, the second model trended to explain more variance than the first model; Δ*R*^2^_adj_ = 0.02, *F*(1, 105) = 2.92, *p* = 0.090; Table 3.

**Table 3:** Complementary switch proportion regression results for testing H2: RT (all participants).

Variable	Model 1				Model 2			
	Estimate	*SE*	*|t|*	*p*	Estimate	*SE*	*|t|*	*p*
Constant	0.175***	0.015	11.70	8.61 × 10^–21^	0.174***	0.015	11.80	5.75 × 10^–21^
**|**RT_K-cued Trials_—RT_M-cued Trials_|	− 0.008	0.015	0.56	0.58	− 0.010	0.015	0.70	0.49
RT_Switch Trials_—RT_Repetition Trials_					− 0.026	0.015	1.71	0.09
*R*^2^_Adj_	− 0.01				0.01			
Δ*R*^2^_Adj_					0.02			

Predictors are based on Block 1, DV on Block 2 trials, compare Fig. 2. The variance inflation factor for both Model 2 predictors is 1.006.

***p < 0.0001.

**Figure 5:**
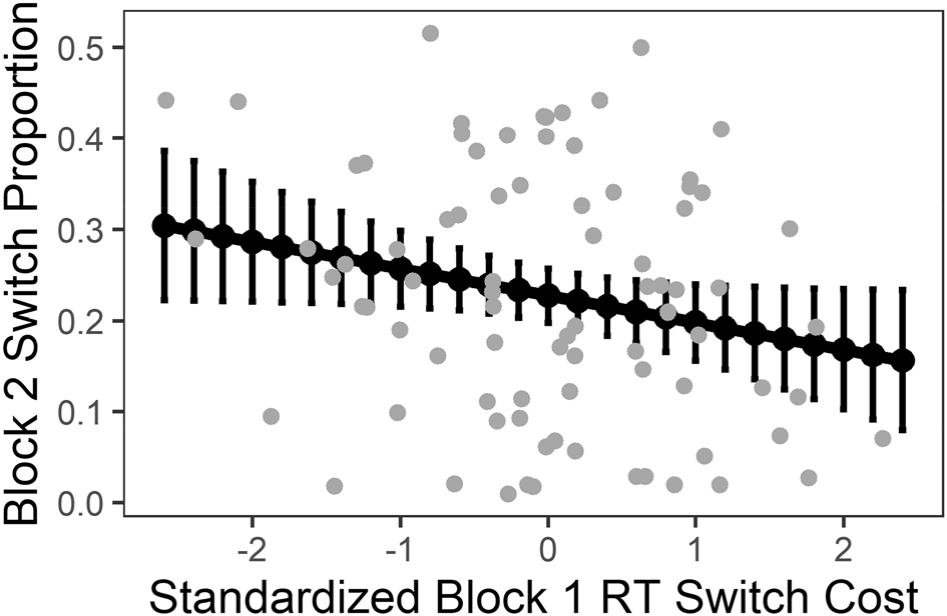
Block 2 switch proportion as predicted by block 1 RT switch costs. Note: the figure depicts Block 2 switch proportion as predicted by standardized Block 1 RT switch cost when holding Block 1 performance differences (**|**RT_K-cued Trials_—RT_M-cued Trials_|) constant. Error bars depict 95% confidence intervals. Gray points represent single participants. The underlying model is based on participants with at least one switch (switch proportion > 0) during Block 2.

We also replicated the analysis omitting participants with 0 switch trials. In this case, absolute RT differences between K-cued and M-cued Block 1 trials again did not significantly predict Block 2 switch percentage, *R*^2^_adj_ = 0.02, *F*(1, 82) = 2,93, *p* = 0.09. However, when adding RT switch costs between K-cued and M-cued Block 1 trials as a predictor, a significant amount of Block 2 switch proportion variance could be explained, *R*^2^_adj_ = 0.06, *F*(2, 80) = 3.50, *p* = 0.03; Table 4; this second model also explained more variance than the first model; Δ*R*^2^_adj_ = 0.034, *F*(1, 82) = 3.96, *p* = 0.0499, compare Table 4. Specifically, a one standard deviation increase in Block 1 RT switch costs was associated with a 3 percentage point drop in Block 2 switch proportion, when holding absolute Block1 RT differences between K-cued and M-cued trials constant, Fig. 5.

**Table 4:** Complementary Switch Proportion Regression Results for Testing H2: RT (participants with Block 2 switch proportion > 0).

Variable	Model 1				Model 2			
	Estimate	*SE*	*|t|*	*p*	Estimate	*SE*	*|t|*	*p*
Constant	0.232***	0.015	15.21	1.84 × 10^–25^	0.231***	0.015	15.45	9.81 × 10^–26^
**|**RT_K-cued Trials_—RT_M-cued Trials_ |	0.034	0.020	1.71	0.09	0.031	0.020	1.57	0.12
RT_Switch Trials_—RT_Repetition Trials_					− 0.03	0.015	1.99	0.05*
*R*^2^_Adj_	0.02				0.06			
Δ*R*^2^_Adj_					0.03			

Predictors are based on Block 1, DV on Block 2 trials, compare Fig. 2. The variance inflation factor for both Model 2 predictors is 1.007.

***p < 0.0001; * *p* = 0.0499.

We additionally replicated the quasi-binomial logistic regression with RT instead of RT* as DV. We omit reporting the full results because results are highly similar to the regular OLS regression. In short, when including all participants in the analysis, deviance of the full model with two predictors trended to be lower than the deviance of the model with one predictor (i.e.,** |**RT_K-cued Trials_—RT_M-cued Trials_|); Step 1: df = 106, *χ*^2^ = 2171, Step 2: df = 105, *χ*^2^ = 2114, Δ*χ*^2^ = 56.6, *p* = 0.07. When only including participants with a Block 2 switch proportion > 0, the trend strengthened: Deviance of the full model with two predictors was lower than the deviance of the model with one predictor (i.e.,** |**RT_K-cued Trials_—RT_M-cued Trials_ |); Step 1: df = 81, *χ*^2^ = 1008.9, Step 2: df = 80, *χ*^2^ = 963.1, Δ*χ*^2^ = 45.9, *p* = 0.04. In sum, the relevant effects of the quasi-binomial logistic regression mirrored the effects of the OLS regression with slightly lower associated *p*-values.

### Exploratory data analyses

Exploratory data analyses are focused on providing additional insight into (1) CS switch costs and (2) the relationship between switch costs and actual switching behavior in a free choice setting. In other words, (1) is exploring H1 further ands (2) is exploring H2 further.

*(1) Switch costs in Block 2* In Block 1, switching CSs induced RT costs of about 103 ms. Do these costs transfer over to Block 2? Given the differences between both Blocks, constant switch costs do not seem likely. First, there might be learning effects, leading to diminished switch costs. Second, the abundant switching in about 50% of trials during Block 1 dropped to occasional switching in about 17% of trials in Block 2. More infrequent switches might lead to increased costs. Third, participants did not need to decide which CS to use in Block 1. This situation changed in Block 2 and might either induce additional costs in both repetition and switch trials or, if choice processing is sometimes omitted during repetition trials, induce more costs in switch trials. Please note that we referred to RT instead of RT* switch costs in the present paragraph because the RT* switch cost effect was shown to exclusively depend on RT in the present study. Statistical significance-wise, the explored effect also holds when using RT* as DV.

**Figure 6:**
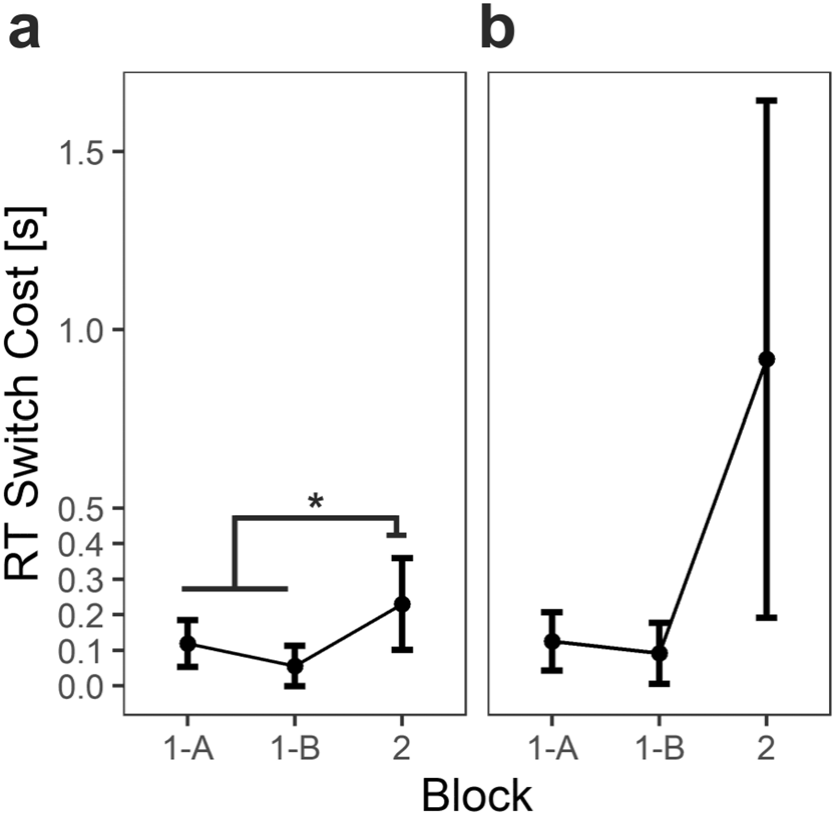
Switch costs across blocks. Note: data from participants with at least 8 (**a**; n = 67) or less than 8 (**b**; n = 41) Block 2 switch trials. Block 1 switch costs stem from a cue-based design whereas Block 2 switch costs stem from a free choice design and are harder to interpret and possibly confounded (for more details, please see section Exploratory Data Analyses: Switch Costs in Block 2). Error bars depict 95% CIs. *p = 0.04.

For the present analysis, we determined switch trials based on arrow key presses (since no cues were used in Block 2). To avoid overly noisy switch cost estimates, we omitted all participants with less than eight switch trials in Block 2, which leads to inclusion of 67 out of 108 participants for the analysis. A dependent *t*-test comparing Block 1 with Block 2 indicated a substantial increase in RT switch costs from 87 to 230 ms in the analyzed subsample of 67 participants; *t*(66) = 2.08, *p* = 0.04; Fig. 6a. Note that switch costs are slightly different here than stated in H1 because (1) RT instead of RT* was used, (2) because switches in the present analyses needed to be defined slightly differently than for H1 due to the design differences between blocks, i.e. by switches in arrow key presses between trials instead of cue-based switches, and (3) the sample was different. For full disclosure, we also report descriptive results for the remaining subsample of 41 participants; Fig. 6b. A descriptively larger but also noisier effect was present for participants with less than eight Block 2 switches (increase from 108 to 917 ms RT switch costs).

In sum, what does this tell us about Block 2 switch costs? First, the descriptive decline in switch costs from Block 1-A to Block 1-B that might represent some learning does not overtly continue in Block 2. Instead, second, switch costs rise. Correlating the proportion of Block 2 switch trials with Block 2 switch costs gives us a first hint that fewer switches might increase switch costs; Pearson’s *r* = − 0.21, *t*(65) = 1.71, *p* = 0.09. However, an alternative explanation would be that participants with high Block 2 switch costs simply avoid switching frequently. Interestingly, Block 1 and Block 2 switch costs do not correlate (*r* = 0.07, *t*(65) = 0.61, *p* = 0.55). Thus, even though Block 1 switch costs mildly predict Block 2 switch rate, Block 2 switch costs are largely independent from Block 1 switch costs. A likely reason is that in Block 2, the choice-related processing in Block 2 explains the major part of the switch costs.

Lastly, we have to admit that with the present data, and possibly with most paradigms, switch costs stemming from free choice settings are hard to interpret. Nevertheless, we want to stretch the point that free choice settings are closer to real-life CS choice behavior than cued settings, which is why we deem them critical to consider. In that vein, the present analysis suggests that cued switch costs might underestimate CS switch costs as encountered in real-life settings.

*(2) Why do some people not switch in Block 2?* Our present results suggest a small but reasonable relationship between switch costs and free choice switch frequency: When including all participants into the analyses, RT switch costs *trended* to increase the amount of explained Block 2 switch proportion. This trend manifested, based on the admittedly questionable 0.05 alpha level, when excluding participants with zero switches. One underlying reason might be of statistical nature, i.e. reducing the non-normality of the error variance. But apart from statistical reasons, *zero switchers* might be qualitatively different from participants who switch. Here, we explore this assumption with the data at hand.

Firstly and unsurprisingly, when asked how frequently participants thought about the decision between the mental and keyboard CSs in Block 2, the 25 zero switchers frequently answered that they “chose once which CS to use at the very beginning of Block 2” instead of for “each”, “most” or “some” of the 144 problems encountered in Block 2 (12 out of 25 zero switchers [48%] vs. 11 out of 83 switchers [13%]). Intuitively, such a one-time decision would especially make sense if trial-by-trial switching costs were especially high during Block 1. This was however not the case: the 25 zero switchers had a 105 ms Block 1 RT switch cost which is highly similar to the 103 ms switch cost of the remaining 83 participants (the 12 zero switches who reported to have decided once at the beginning also had similar costs of 111 ms). Thus, many zero switchers seemed to have consciously made a decision once at the beginning instead of more frequently throughout Block 2. It is unclear why they made this decision.

To answer this question, we report zero switchers’ answers to an open question on why they chose CSs the way they did in Block 2. Interestingly, none of the 25 zero switchers mentioned switch costs. Most prominently, 11 participants reported mental rotation to be “more effective”/”easier”/”faster”/”more accurate”, or “better”. Furthermore, 1 participant mentioned that manual rotation was “easier”, 1 that manual rotation was less “effortful” and he felt “tired”, 1 used manual rotation to double-check mental rotation, 2 found that mental rotation felt “more natural” (1 of them also reported mental rotation to be easier), and 10 gave no or no meaningful answers. We conclude that zero switchers mostly had performance in mind when deciding for zero switches, which does not qualitatively set them apart from the remaining sample. This performance-related reasoning is backed by Block 1 performance data: When comparing the absolute performance differences between K-cued and M-cued Block 1 trials, zero switchers had 241 ms larger absolute RT differences than switchers; *t*(106) = 2.58, *p* = 0.01. No differences were apparent regarding accuracy; *t*(106) = 0.17, *p* = 0.87. One possible conclusion is that zero switchers are not *qualitatively* but *quantitatively* different from switchers in that, for them, performance differences between CSs were larger to a degree where switching costs might be negligible.

*Final remarks* Our exploratory data analyses made most clear that human problem solvers can use different CSs in a fluid—i.e., not in a black-or-white—manner. When asked how often they used manual and mental rotation in parallel for the very same problem, more than half answered “always” or “frequently” (12% always, 42% frequently). The remaining participants answered “rarely” (28%) or “never” (18%). Such variability in parallel processing preference was also found with the task switching paradigm could be related to individual styles of cognitive control^36^. That our problem solvers used CSs in a fluid manner was most prominent in Block 2. In Block 2, participants frequently decided to switch *within* trials. Specifically, about half (i.e., 40 participants) out of all participants with at least one switch in Block 2 (i.e., 83 participants) reported to mix CSs *within* a trial (17 provided no meaningful answer). In an open answer format, these 40 participants describe in one way or another that they would first try to find the answer using the mental CS and if that “failed”, or they were “not completely certain” about the answer, or realized that the present problem was “hard”, or “the answer was not obvious”, they would then use the manual CS. In other words, many participants established a sequential heuristic that should eliminate some decision costs. Nevertheless, substantial switch costs were present in Block 2, which suggests a certain robustness of the CS switch cost effect that had been found during Block 1 with the cued design.

We speculate that switch costs in a free choice setting like in Block 2 are related to whether participants use CSs in isolation, in parallel, or in sequence. For example, in a trial in which participants followed a sequential approach and failed to find a solution mentally first, they will then continue using manual rotation. Will there be switch costs in the following trial if they again start with mental rotation? Are there even asymmetric costs because manual rotation might always be accompanied by some mental rotation but not vice versa—and thus, switches from mental to manual might be more taxing? Further research will be able to illuminate how the different ways people employ CSs in a specific trial affects switch costs in free choice settings.

## Discussion

The present study confirms that alternating between different strategies to solve similar problems can be costly. Costs were shown for cued switches (~ 100 ms; H1) and for freely chosen switches (> 200 ms; Exploratory Data Analysis: Switch Costs in Block 2). The cued CS switch costs are of similar size as reported for cued task switch costs^34^. Present results also mildly suggest that participants adjusted their CS switching behavior in a free choice setting based on these costs (H2). In other words, results suggest individual differences in “switch proficiency”, which in turn affect switching behavior. This result resembles the relationship between individual task switch costs and switch rate in voluntary task switching (Kool, et al., 2010; Mayr & Bell, 2006) despite procedural differences to previous task switching studies such as much lower RSIs and no instruction to use both tasks equally often.

We conclude three things. First, individual switch costs as found in voluntary task switching are also relevant in similar settings in which performers need to decide between different CSs. Second, individual switch costs are relevant for choice processes in more applied settings, given that the present study featured a rather long RSI and actual free choice between CSs—in contrast to instructions to engage in both of two tasks equally often, as in voluntary task switching. Third, individual switch cost seems to be only a minor contributor to both task and CS switch rate. Even when only looking at performers who *did* switch CSs in our free choice setting, the impact of individual switch costs was modest. In real world settings, many participants might not switch at all, which could, for example, be due to CS performance differences^28^ rather than switch costs. Interestingly, switching CSs was costly even though—and unlike the costs observed in task switching^12,13^—the overarching task/goal stayed identical throughout all trials. Furthermore, switching was costly even though participants reported frequent parallel processing and the used CSs (manual and mental rotation) might rely on overlapping mental operations^7^, which should lead to a reduction of CS when compared to task switch costs.

CS switch costs are relevant, foremost, because an apprehension of CS switch costs is relevant for an efficient use of cognitive environments as, for example, in cognitive offloading [compare 6, 8, 17, 50]. Also, when different CSs exhibit similar performances regarding accuracy and RT, switch costs render repetition of one CS more efficient than switching between CSs. Previous research on CS choice incorporated a delay before a screen would allow access to a computer-based CS ^e.g.,^^14,50^ or a physical distance that needed to be overcome by walking before gaining access to a computer-based CS^51^ to alter a specific CS’s performance. Participants adaptively adjusted their CS choice behavior based on these performance alterations. Switch costs can be interpreted from a similar point of view. Similar to worsening access to a CS, switch costs degrade a CS’s performance. Specifically, switch costs selectively degrade the performance of currently unused CSs. A corollary of this observation is that context-independent descriptions of a CS’s performance are not sufficient to exhaustingly guide efficient CS choice. Instead, previous CS choices need to be additionally taken into consideration when evaluating a CS’s performance.

Similarly, context-independent CS performance is likely not enough to explain naturalistic CS choice behavior: Present data suggests that participants alter CS switch rate based on RT switch costs. The underlying reason might be to minimize objective time ^e.g.,^^14^, subjective time^52^, or cognitive effort. In other words, time might not be the only underlying reason. Instead, task switches might be frequently avoided because they evoke cognitive effort via demands on executive control^31^ and cognitive effort is linked to negative affect when conflict resolution is slow^53,54^, which is aversive. It should also be kept in mind that the relationship between choice costs and switch rate found in the present investigation is small and was only found for participants who *did* switch. One possible explanation is that extreme preference (i.e., exclusively sticking to one CS) is more frequently caused by differential CS performance than by switch costs because of their larger effect size for some individuals: Switch costs accounted for about 100 ms whereas absolute RT differences between manual and mental rotation were about 240 ms for “zero switchers” (compare: Why do some people not switch in Block 2?).

Alternatively, extreme strategy preference might also have its roots in an *Einstellung*, which could be circumscribed as a form of cognitive inertia that promotes the repetition of a frequently used previous way of solving a task^55^. However, participants were able to acquire ample experience with *both* strategies in the forced choice part of the present study, such that an *Einstellung* would only be able to explain extreme strategy preference if the *Einstellung* was created at the beginning of the free choice block rather than during the forced choice block.

### Limitations and future research

The maybe most relevant but also most exciting limitation of the present study pertains to generalizability. Are all CS switches costly? Or, conversely, is it possible to use CSs that harmonize in a way that eliminates switch costs? Are there only RT-based CS switch costs—as in the present study—or are there situations with accuracy costs as well, as has been reported for task switching^13^? And, after all, are there other causes for repetition or switching beyond performance and cognitive effort, such as affective preferences for one CS over another?

We are also curious about the origins of the switch costs, which are known to be manifold during task switching^13^. Similar to previous studies on voluntary task switching^29^, we found costs that were independent of choice processes during blocks in which the choice process was replaced with a cue. These costs increased once the cue vanished and participants had to choose on their own, suggesting higher switch costs in settings without cues. Transferred to a real-life context, this could mean that guided strategy switches are less costly than self-directed switches. Alternatively, higher switch costs in free choice trials of the present paradigm might stem from the fact that during cued trials, participants were able to prepare the respective CS 500 ms before the stimulus started (i.e., when the cue emerged; compare Fig. 1b, c). During free choice trials, a portion of participants might not have used this window for CS preparation but instead waited for the stimulus to decide which CS to use, which is similar to late in comparison to early deciders in previous research^56^. An overall higher workload due to constant decision making might also have contributed to more pronounced switch costs.

Switch costs with different origins might also affect switching behavior in different ways. More cognitive switch costs (e.g., activating different rule sets or similar) might lead to more repetitions than more physical switch costs (e.g. walking a certain distance to use a CS) or idle time without cognitive or physical costs. The underlying idea is that human problem solvers might be especially willing to avoid cognitive effort^31^. However, it has been shown that for voluntary task switches, physical costs factor in as well^57,58^. Physical switch costs are present to some degree in the present paradigm (manual rotation necessitates pressing a key that needs preparation) but are likely more pronounced in more physically engaging situations like, for example, when walking to a computer to gain access to a CS ^compare^^51^.

Lastly, to understand the relationship between switch costs and free choice task switches, future studies should avoid task and strategy properties that discourage task switching irrespective of switch costs. Here, 25 participants did not switch at all during the free choice block. A recent study suggests that introducing a calibration procedure that eliminates performance differences between strategies would have likely eliminated most of these zero switchers and thus increased the present study’s power^28^. On the other hand, the present study –which was conducted with uncalibrated strategies– might be closer to reality in which strategies rarely feature identical performance.

## Conclusion

The present study suggests that switching between different cognitive strategies to solve the same task is costly. Such costs, here indicated by slower responses, are a conceivable reason for the tendency of human agents to stick longer rather than shorter to one strategy. Thus, switch costs can stand in the way of a truly dynamic use of a variety of cognitive strategies for the same problem. From a problem solver’s perspective, switch costs should therefore be considered when choosing between different strategies. From a designer’s perspective, analogously, switch costs should also be considered when creating environments that help people think.

### Supplementary Information

Supplementary Information.

## Data Availability

The datasets generated and analyzed during the current study are available in the OSF repository at https://osf.io/cszgw. The experiment was preregistered at https://osf.io/uaex9.
