# Supplementary material for: Switching between different cognitive strategies induces switch costs as evidenced by switches between manual and mental object rotation
Source: Sci Rep. 2024 Mar 14;14:6217. doi: 10.1038/s41598-024-56836-2 (PMC10940645; doi:10.1038/s41598-024-56836-2)
Supplement: Supplementary file 1 — Supplementary Information. [file 41598_2024_56836_MOESM1_ESM.pdf]

Supplemental Materials

Complementary Analysis to H1

These analyses allow tearing apart the impact of switches on RT from the impact on accuracy, since the analysis reported in the main manuscript only features the combined measure RT*.

Table S1
RT ANOVA Results (Block 1 Trials only)

	DF1	DF2	*F*	*p*	η_G^2
Angle ***	1	107	180.2	1.1×10^{-24}	.033
Cue ***	1	107	81.9	7.1×10^{-15}	.070
Switch ***	1	107	40.0	6.0×10^{-9}	.004
Angle x Cue ***	1	107	15.6	.0001	.002
Angle x Switch	1	107	1.0	.320	< .001
Cue x Switch	1	107	.4	.507	< .001
Angle x Cue x Switch	1	107	.1	.771	< .001

Notes. *** $p < 0.001$

Table S2
Accuracy ANOVA Results (Block 1 Trials only)

	DF1	DF2	*F*	*p*	η_G^2
Angle ***	1	107	110.2	3.8×10^{-18}	.065
Cue ***	1	107	121.8	2.3×10^{-19}	.166
Switch	1	107	< .1	.868	< .001
Angle x Cue ***	1	107	53.3	3.6×10^{-8}	.023
Angle x Switch	1	107	.1	.746	< .001
Cue x Switch	1	107	.7	.422	< .001
Angle x Cue x Switch	1	107	.2	.650	< .001

Notes. *** $p < 0.0001$

Also note that—as indicated by the significant cue main effects (Table S1, Table S2) — mental and manual rotation entailed different performances. Specifically manual rotation was more accurate ($M_{\text{K-cued trials}}$ = 93.2%; 95% CI = [92.4 – 94.0]) than mental rotation ($M_{\text{M-cued trials}}$ = 85.5%; 95% CI = [83.8 – 87.1]). Conversely, manual rotation was also slower ($M_{\text{K-cued trials}}$ = 3530 ms; 95% CI = [3380 – 3680]) than mental rotation ($M_{\text{M-cued trials}}$ = 3100 ms; 95% CI = [2970 – 3240]).
